# Aggression shapes the gut microbiome; a study in rats

**DOI:** 10.1371/journal.pone.0312423

**Published:** 2024-10-22

**Authors:** Anna Voulgari-Kokota, Joana Falcao Salles, Regien G. Schoemaker

**Affiliations:** 1 Groningen Institute for Evolutionary Life Sciences (GELIFES), University of Groningen, Groningen, The Netherlands; 2 Laboratory of Microbiology, Wageningen University, Wageningen, The Netherlands; University of Ghana, GHANA

## Abstract

The gut-brain axis is regarded as a bidirectional communication system that integrates signals from the gut microbiome into behavioral aspects and vice versa. The aim of the present study was to investigate the gut microbiome-behavior interaction in relation to aggression. For that, male rats from a group-housed colony were individually housed with a female to become territorial. Next, a coping strategy was assigned to them, by evaluating their aggression levels against an intruder, during the Resident-Intruder test (RI). To investigate if their microbiome would change as a consequence of the developed coping strategy, fecal samples were collected before and after the RI test. We found that the relative abundances of Ruminococcaceae UCG-5 and Gram-negative bacterium *cTPY-13* in rats sampled before the RI test were negatively correlated with the aggression that was demonstrated during the RI test. After the RI test, several bacterial taxa could be assigned to each coping strategy, with *Clostridium sensu stricto 1* being strongly associated with less aggressive rats and higher abundances of *Bifidobacterium*. Furthermore, the family of Lachnospiraceae was not only associated with more aggressive rats, but functional prediction analysis found it to be the main contributor of betaine reductase; an enzyme catalyzing betaine production that was indicative of aggressive rats. This amino acid derivative, which has been connected with higher energy and testosterone levels, could potentially explain the connection of Lachnospiraceae with demonstrated aggression. Overall, our data revealed that the gut bacterial communities are responsive to the imposed social challenge of building and defending territoriality in co-habitation with a female. At the same time, predisposing microbiome characteristics may have predictive value for the development of a coping strategy.

## Introduction

The gut-brain axis is a bi-directional communication system, that integrates neuronal, hormonal and immunological signaling between the gut and the brain [1, 2]. The gut-brain axis is comprised of neural pathways, such as the Enteric Nervous System (ENS), the vagus, sympathic and spinal nerves, and humoral pathways, including cytokines, hormones, and neuropeptides, as signalling molecules [3]. Hence, a direct relation between the gut microbiome and host behavior has been reported [4]. More specifically, a gut microbiome-aggressive behavior relation is supported by studies in mice [5], hamsters [6], laying hens [7], and dogs [8, 9], and has been recently reviewed [10, 11]. What is more, recent studies have reported that changing the microbiome could alter the coping style of mice [5], while dietary interventions could affect aggressive behavior, as reviewed by Tcherni-Buzzeo [11], underpinning the dynamic bi-directional character of the gut brain behavior interaction.

Groninger wild type (GWT) rats are known for their broad inter-individual variation of behavioral responses. Regarding coping strategy in the resident intruder test (RI), where a resident rat gets challenged by the introduction of an intruder [12], three subtypes were distinguished–low aggressive or reactive, medium aggressive or intermediate, and high aggressive or proactive animals [13]–which are indicated as animal “personalities” [14]. Once established, these coping strategies may be extended to other challenges, such as defensive burying, performance in the Porsolt forced swim test, and the orientation reaction to sudden silence, but not in emotional reactivity tests, such as open field exploration and elevated plus maze, as reviewed by de Boer et al. [13, 15]. Moreover, coping strategies seemed reflected in distinct physiological characteristics, with proactive rats appearing more sympathetically driven, based on neuroendocrine markers, autonomic nervous system markers, and neurobiological parameters [13]. This difference resulted in differential susceptibility to diseases, including autoimmune disease, cardiovascular disease, obesity/metabolic syndrome, as well as substance use disorder, and stereotypes/compulsivity [13]. Although this rat strain is extensively studied with regard to aggressive behavior, not much is known about their gut microbiome. Since these rats are bred in our facility, rats could be studied in a naïve state regarding the development of a specific coping strategy.

Pilot studies in our group indicated a rather uniform microbiome under breeding conditions, but significant differences in rats challenged towards developing a specific coping strategy (unpublished data). The aim of the present study was to further investigate the bidirectional gut-microbiota-behavior interaction in the GWT rat. For that, we assigned personalities to rats by performing the RI test for each individual included in the study. We characterized their gut bacterial communities before and after RI training and testing to evaluate whether the gut microbiota provides a predisposition for the development of a coping strategy and/or whether a coping strategy would be reflected in an altered gut microbiota. We hypothesized that the microbiome is relatively uniform in the breeding situation but changes as a consequence of developing a coping strategy in the challenging RI test. We aimed to reveal bacterial taxa recurrently present in the GWT rat gut and others associated with specific personalities. Finally, we aimed to assess the degree to which the gut microbiotas shift through the RI experience in terms of diversity and composition.

## Materials and methods

Male Groninger Wildtype rats (GWT) (age >180 days) were collected from our breeding colony (University of Groningen). Until the start of the experiments, rats were group-housed with 5–6 rats of the same sex per cage, under controlled climate conditions (temperature of 20±2°C and humidity of 50±10%). They were provided with standard rat chow (Hope Farms, Woerden, the Netherlands) and water *ad libitum*. All experiments were approved by the local animal committee of the University of Groningen and the National Animal and Welfare Committee of the Netherlands (CCD) and animal handling took place in accordance with the ARRIVE guidelines for in vivo Animal Research. All experiments were performed on rats involved in other studies as spin-offs without additional discomfort for the rats and did not include any procedure that would be harmful to the them. For the present study, fecal samples were collected from rats after social encountering with other animals, to aseess their gut microbiome, as described in the next subsection. Collection of fecal samples does not involve discomfort to the animals. The study included 24 rats, however one rat that lost 5% of body weight for three days in a row was excluded, while one rat died on the second day of the experiment out of unknown cause. The overall experiment including training, testing, as described in the following paragraph, and sample collection lasted two weeks and rats were monitored and weighed daily.

### Resident intruder test

To assign a personality to the rats with regard to aggression, we performed the resident intruder test (RI) based on the resident intruder paradigm as described by Koolhaas *et al*. [12]. Initially, male rats were collected from the group-housed breeding facility and housed individually in large cages (0.5 m^2^) with a female rat on reversed light: dark cycle (12:12) with the lights out at 10:00 am. The female rats had ligated oviducts to maintain hormonal activity without pregnancy. After at least one week, the male rats had developed territoriality. Then, training and testing started. At the moment of training/testing, the female rat was temporally removed, and an unfamiliar male rat (slightly lighter than the resident) was introduced into the cage. More specifically, training was performed on three consecutive days, with one different intruder being introduced at each time. Behavior was recorded at all training sessions. Intruders were removed shortly after the first attack of the resident, with a maximum of 10 minutes, and after that the female rats were returned to the cage. After three training sessions, aggressive behavior had been stabilized [12]. One day after the last training session, the actual test was performed, by leaving the intruder in the cage for 10 minutes. Aggressive behavior was measured by attack latency, number of attacks, and time spent on offensive behavior. The outcomes of these parameters were ranked, and the average rank was used as the overall aggression score. Based on the aggressive behavior in this latter test, rats were divided into tertiles: highly aggressive, medium aggressive, and low aggressive, as measures for proactive, intermediate, and reactive coping, respectively.

In total, 24 GWT male rats were included in the RI training and testing. From all individuals, fecal samples were collected directly by gentle massage twice, before and after the RI test. The first samples were collected immediately after selection from the breeding colony, and the second samples were collected after the RI test using the same method. All samples were stored at -20°C for later analyses. Samples from three rats were excluded from the final dataset: one died, one lost weight during the experiment and one did not yield sequencing results of sufficient quality, as described in the *Data analysis* paragraph. In conclusion, 21 rats were included in all downstream analyses.

### Fecal sample processing

DNA extraction from the fecal samples was performed with the Qiagen PowerSoil DNA extraction kit (QIAGEN N.V, Hilden, Germany), according to the manufacturer’s instructions. Library preparation and sequencing for bacterial metabarcoding were performed at the Genomics Center of the University of Minnesota using the Illumina MiSeq platform. For every sample, the gene copy numbers for the bacterial 16SrRNA gene were assessed with qPCR. Then, dual-indexing was used to create DNA libraries. The DNA library preparation included purification, normalization, and pooling. Amplicon sequencing consisted of a 2×300 base pairs run for the V4 region of the bacterial 16S rRNA gene (primers: 515F–926R) [16].

### Data analysis

Quality filtering of the sequencing raw data, denoising, and representative sequence picking was conducted with the *DADA2* plugin [17] of the QIIME2 v2023.5 platform [18]. After the representative amplicon sequence variants (ASVs) were picked, sequence alignment was performed using MAFFT [19]. Based on the sequence alignment, an unrooted phylogenetic tree was constructed with Fasttree [20]. The SILVA 138 reference database was used for the taxonomic assignment of the picked ASVs at a confidence level of 0.97. All raw sequencing files have been submitted to the Sequence Read Archive (SRA) under BioProject ID PRJNA1108488.

Data was further analyzed in R 4.2.3. The dataset was filtered to exclude sequences not annotated as bacterial, and one sample that contained less than 1000 reads after filtering was omitted from further analysis using the package *phyloseq* [21]. Alpha-diversity metrics, the total number of ASVs, Shannon index, and Faith’s phylogenetic diversity (PD) were computed with the R packages *phyloseq* [21] and *ape* [22]. Statistical comparison between alpha-diversity values was conducted with non-parametric Wilcoxon rank tests with the R native package. After transforming the composition tables into UniFrac and Bray-Curtis distance matrices, permutational analysis of variance (PERMANOVA) was performed after confirming that the compared groups showed similar beta-dispersity (*betadisper* p>0.05) using the R package *vegan* [23]. Ordinations of samples were conducted with *phyloseq* [21] and visualized with *ggplot2* [24]. Spearman co-efficient non-parametric rank correlations between diversity metrices of taxa with sample metadata were performed and visualized with the packages *GGally* [25], *Hmisc* [26] and *phylosmith* [27]. Assignment of indicative species (IndVal) [28] to each animal personality and Linear discriminant analysis Effect Size (LEfSe) to determine which taxa were most likely to explain differences between personalities were further conducted with the R packages *indicspecies* [29] and *microbiomeMarker* [30], respectively.

Finally, the software *PICRUSt2* [31] was used to predict metagenome functions of the revealed bacterial communities by calculating *MetaCyc* [32] pathway abundances through structured mappings of EC gene families to pathways. Subsequently, the R package *ggpicrust2* was used to analyze the functional profiles derived from *PICRUST2* by conducting differential abundance analysis and correcting p-values for multiple testing [33].

## Results

**Fig 1:**
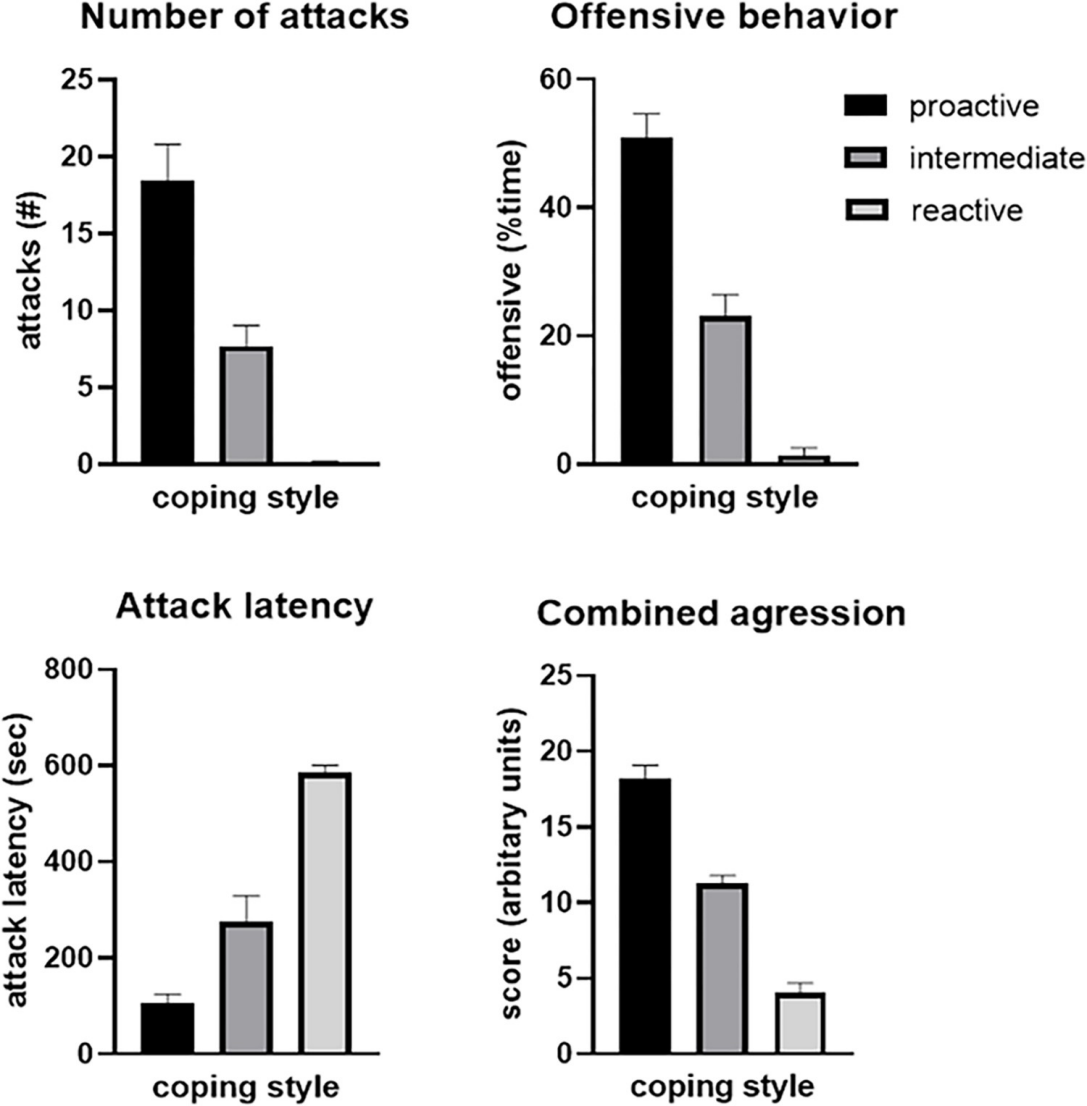
Aggressive behavior in the resident intruder test (RI). Aggression is measured based on attack latency, number of attacks, percentage time spent on offensive behavior, and combined rank-score. Aggression levels are subsequently divided into three coping strategies (proactive, intermediate, and reactive).

All rats included in the study were characterized according to their behavior in the RI test as proactive (n = 7), intermediate (n = 7), and reactive copers (n = 7), according to the levels of demonstrated aggression. Differences in aggressive behavior are reflected in attack latency, number of attacks, and percentage of time spent on offensive behavior (Fig 1).

**Fig 2:**
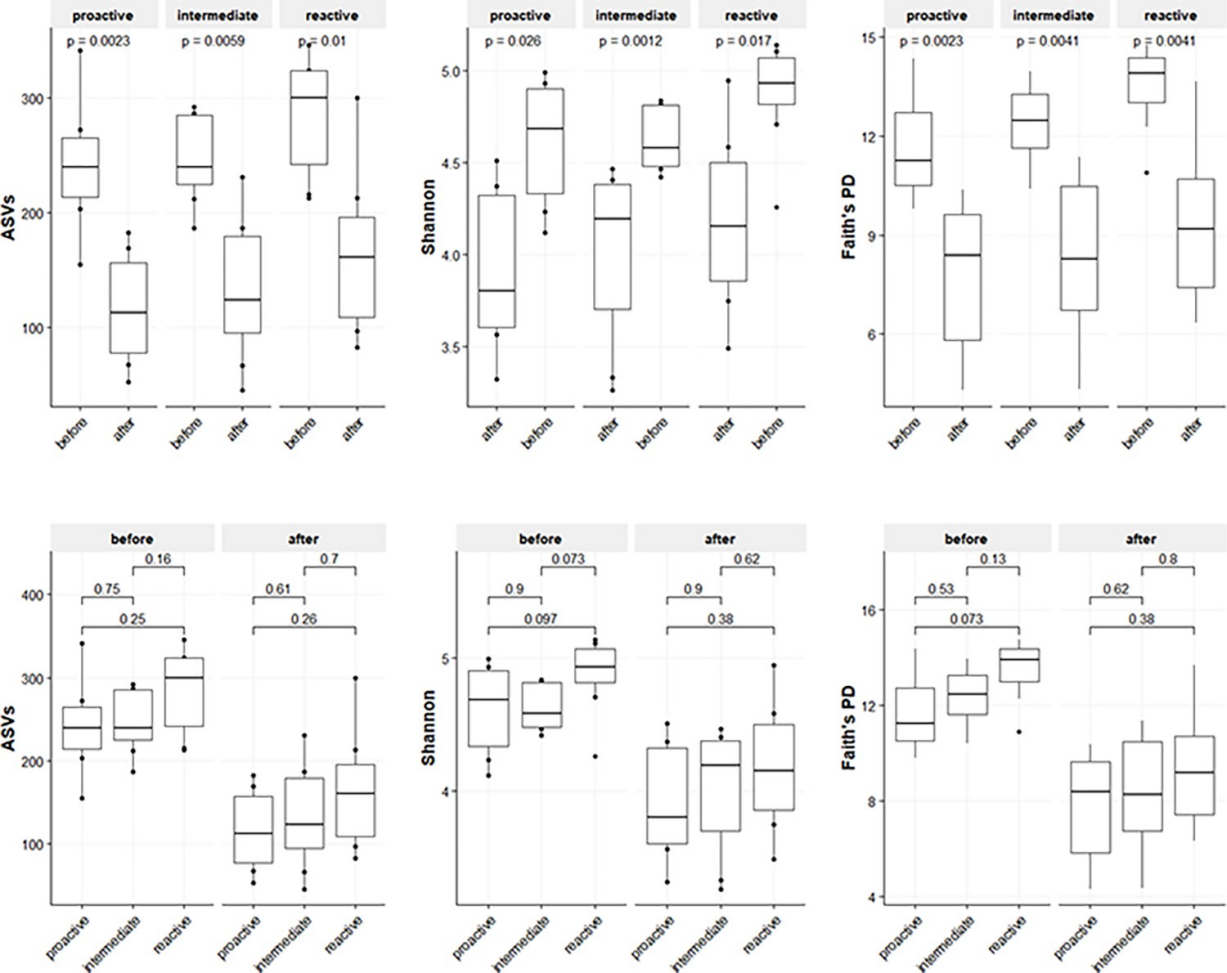
Alpha diversity of the rat gut bacterial communities before and after the resident-intruder (RI) test. Gut microbiota alpha diversity for three personality types: proactive, intermediate, and reactive. Alpha-diversity is estimated with three diversity metrics: the absolute number of Amplicon Sequence Variants (ASVs), Shannon index, and Faith’s phylogenetic diversity (PD). The line in each boxplot represents the median of all values, with the upper limit being the first and the lower limit being the third quartile. P-values are based on pairwise Wilcoxon comparisons.

The GWT rat gut bacterial microbiota analysis returned 1779 unique ASVs after filtering. Comparison of the gut bacterial communities for all rats before and after the RI test showed that the alpha-diversity had significantly shifted after the RI tests for all personality types (Wilcoxon test p<0.01**, for proactive, intermediate, and reactive copers), resulting in less diverse microbiota. No differences in bacterial alpha diversity were observed between groups assigned to different personalities (Fig 2). However, Faith’s PD for the bacterial communities of the rats before the RI test was negatively correlated both with offensive behavior and the number of attacks in the subsequent RI test (Spearman rho = - 0.43, *p<0*.05* and Spearman rho = -0.49, *p<0*.05*, respectively). After the RI test, no significant correlations between the bacterial community alpha-diversity metrics and aggression parameters were found.

**Fig 3:**
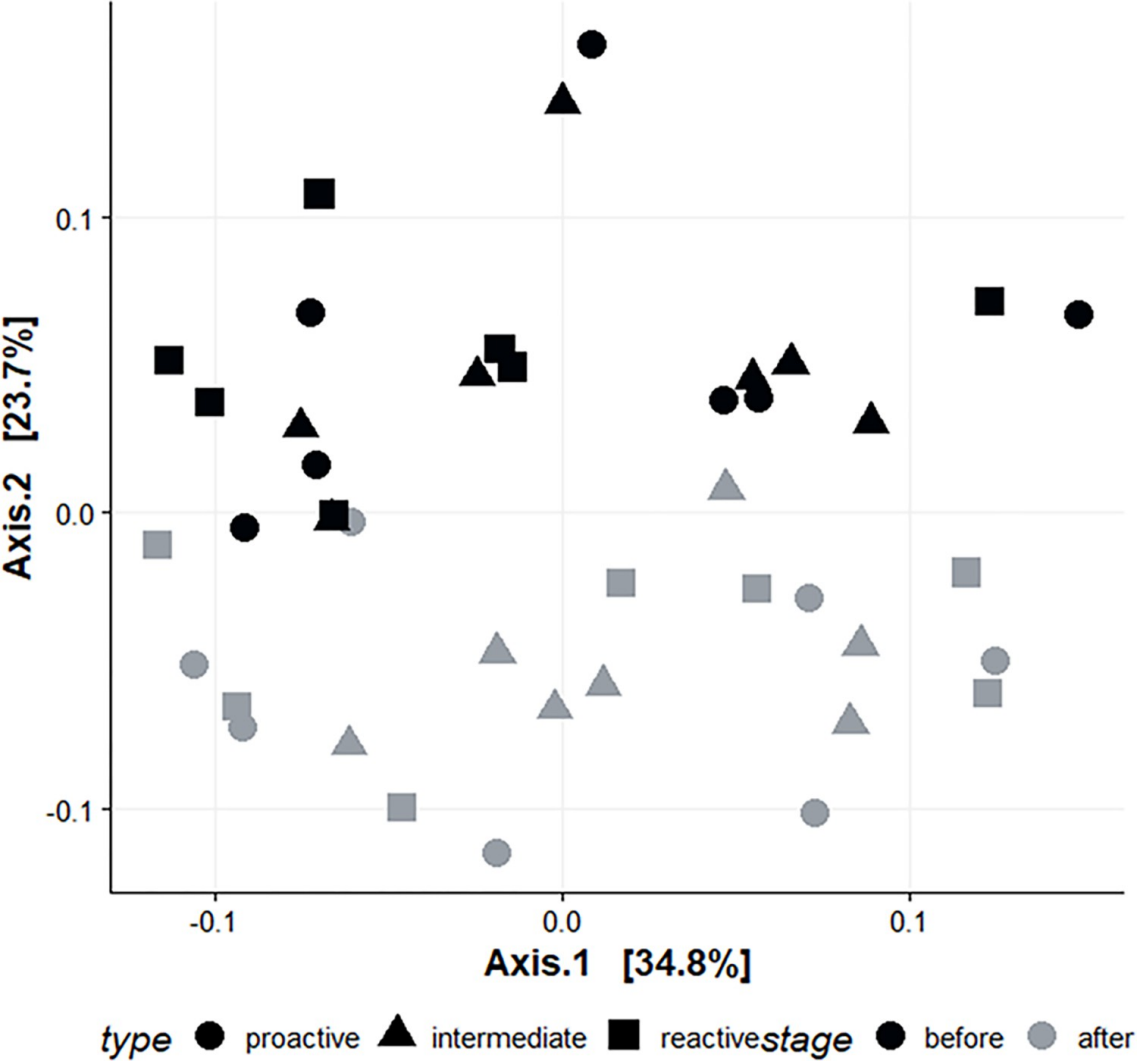
PCoA ordination of the gut bacterial communities for 21 rats based on weighted UniFrac distances. Ordination colors stand for the time the sample was taken (before or after the resident-intruder test), and the shape stands for the personality type of the rats according to their coping strategy (proactive, intermediate, and reactive, as assigned in the resident-intruder test).

The bacterial communities’ beta diversity shifted after the RI experiment (Fig 3). Comparisons of all samples prior to the RI experiment with the ones taken after the RI experiment returned significant PERMANOVA results (R^2^ = 0.16428, *p<0*.001***). A pairwise comparison of the bacterial community composition before and after the RI test revealed that families of higher relative abundance replaced several low-abundance bacterial families. More specifically, Lactobacillaceae, Lachnospiraceae, Oscillospiraceae, and Eubacteriaceae grew in relative abundance (Wilcoxon p values: *<0*.001***, *<0*.01**, *<0*.01**, and *<0*.001***, respectively), while the lowest in abundance families tended to disappear from the microbiome. The bacterial composition of the rat gut microbiota at the family level is illustrated in S1 Fig in S1 File. No significant difference in the overall bacterial beta diversity was found between the three rat personalities neither before nor after the RI test (Fig 3).

**Fig 4:**
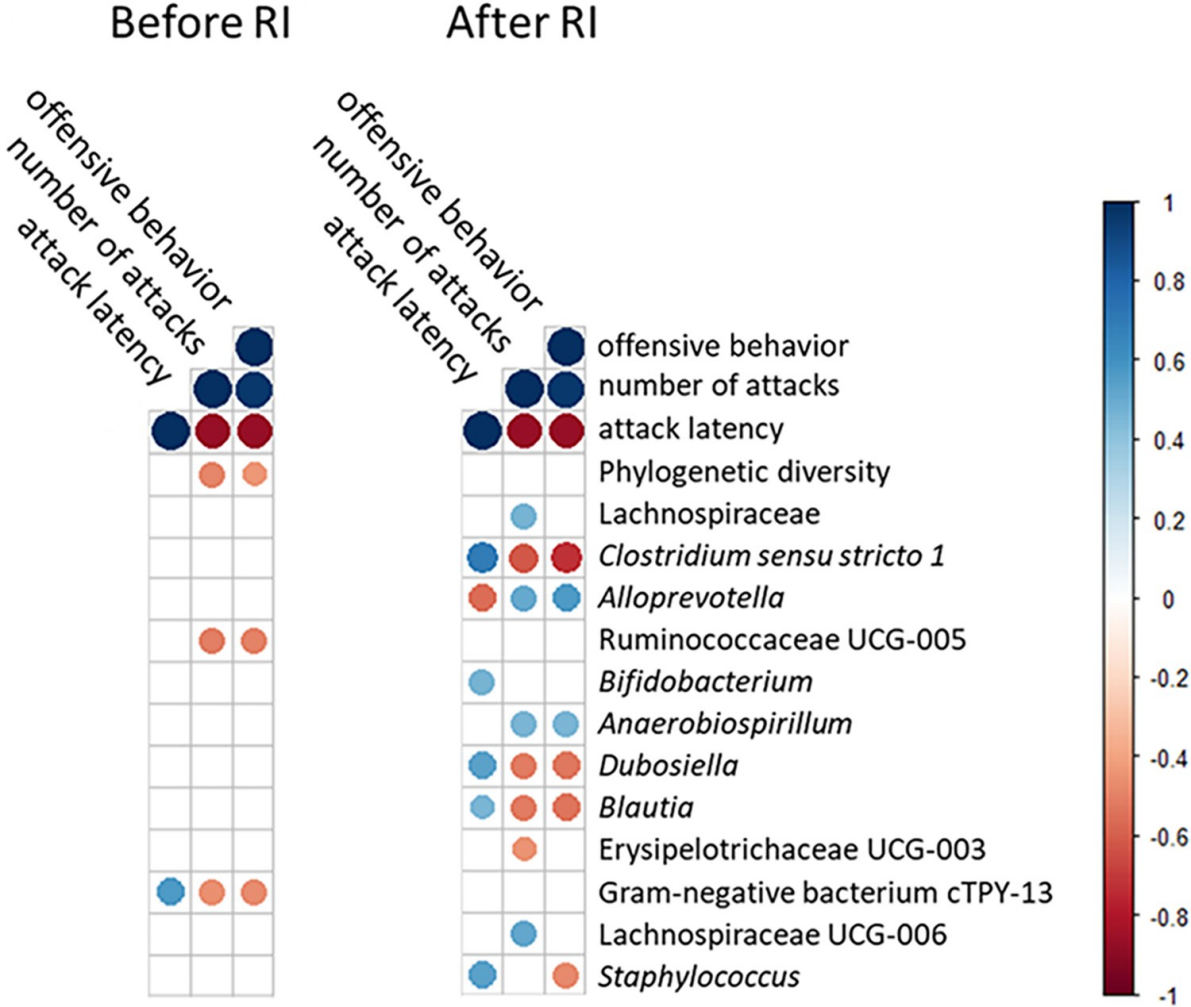
Correlation heatmap between bacterial taxa and aggression parameters. Correlations between the relative abundance of bacterial taxa in the gut of GWT rats before and after the resident-intruder test and three aggression parameters as measured in the test. Correlation values correspond to Spearman’s index and are shown only in the case of statistical significance (*p<0*.*05*).

Before the RI test, the relative abundance of the bacterial family Ruminococcaceae UCG-5 and the Gram-negative bacterium *cTPY-13* were negatively correlated with offensive behavior (Spearman rho = -0.50, *p<0*.05* and rho = -0.46, *p<0*.05*, respectively) and also with the number of attacks in the subsequent RI test (Spearman rho = -0.51, *p<0*.05* and rho = -0.46, *p<0*.05*, respectively). The Gram-negative bacterium *cTPY-13* was also positively correlated with the attack latency in the RI test (Spearman rho = 0.56, *p<0*.01**) (Fig 4). Furthermore, the genus Anaerovorax was associated with reactive rats before the RI test, as shown both with analysis for indicative species for each personality (IndVal = 0.67, *p<0*.01**) and with LefSe (LDA effect size = 2.67, *p*_*adjusted*_*<0*.01**). The same taxa were not associated with any aggression parameters after the RI test was performed.

After the RI test, the genera *Clostridium sensu stricto 1*, *Dubosiella*, and *Blautia* were correlated with all aggression parameters, as measured in the RI test. They were negatively correlated with offensive behavior (Spearman rho = -0.73, *p<0*.001***, rho = -0.52, *p<0*.05*, and rho = -0.54, *p<0*.05*, respectively), negatively correlated with the number of attacks (Spearman rho = -0.61, *p<0*.01**, rho = -0.52, *p<0*.05*, and rho = -0.52, *p<0*.05*, respectively), and positively correlated with the attack latency (Spearman rho = 0.69, *p<0*.001***, rho = 0.53, *p<0*.05*, and rho = 0.45, *p<0*.05*, respectively). The genus *Alloprevotella* was also correlated with all aggression parameters; however positively with aggressive behavior. It was positively correlated with offensive behavior (Spearman rho = 0.57, *p<0*.01**) and the number of attacks (Spearman rho = 0.51, *p<0*.05*), and negatively with attack latency (Spearman rho = -0.56, *p<0*.01**) (Fig 4). The genera *Clostridium sensu stricto 1* and *Blautia* were further associated with reactive rats according to analysis for indicative species for each personality (IndVal = 0.58, *p<0*.01**, and IndVal = 0.65, *p<0*.01**, respectively). LefSe further confirmed the association of *Clostridium sensu stricto 1* with reactive rats (LDA effect size = 3.91, *p*_*adjusted*_*<0*.01**). The same taxa were not associated with any aggression parameters prior to the RI test.

**Fig 5:**
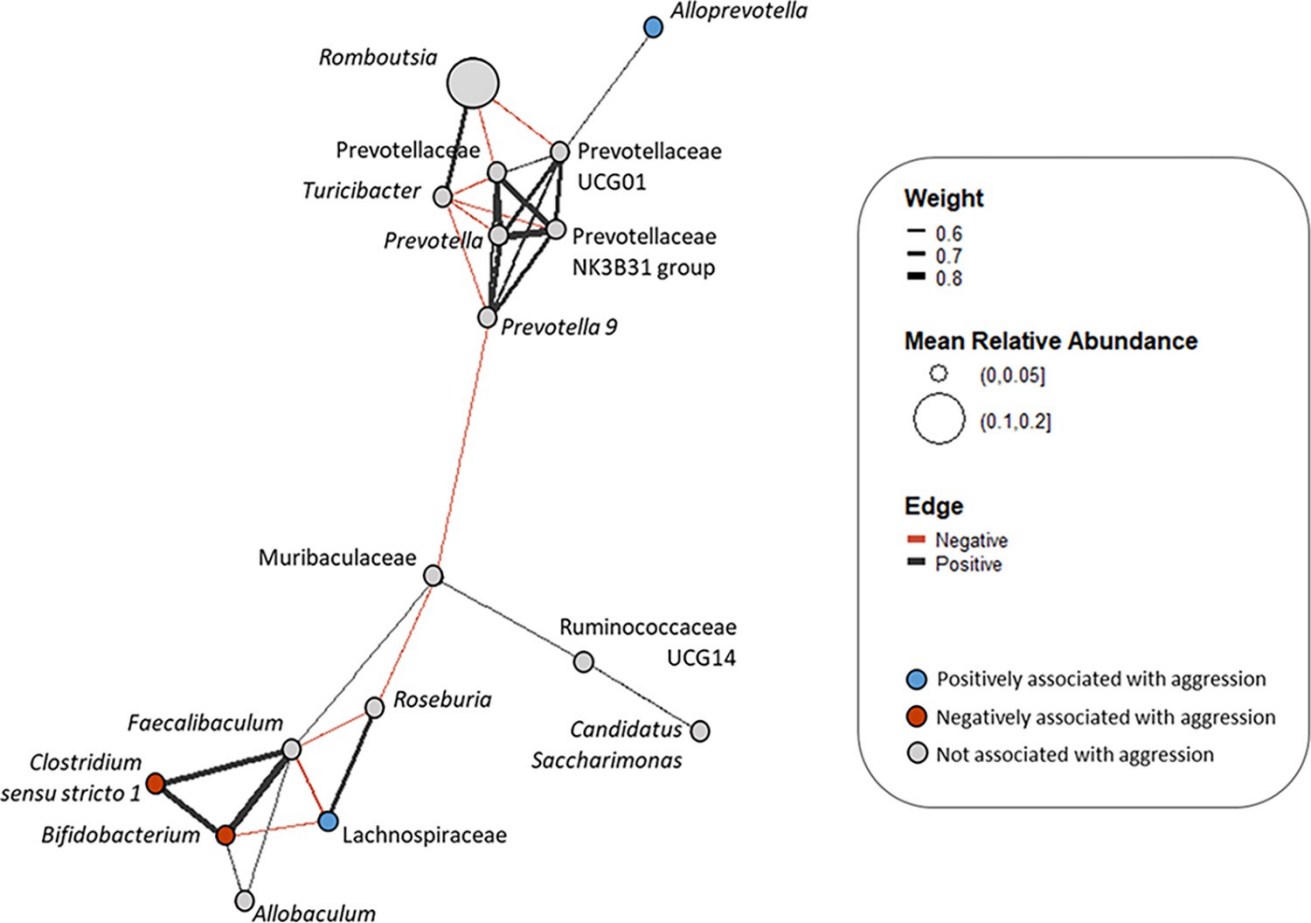
Co-occurrence network of bacterial taxa for all GWT rat gut samples. Associations are based on Spearman correlations if the weight of the association is rho > 0.50 and if the statistical significance has *p<0*.001***. Edge colors represent negative or positive associations, and node colors indicate that the respective taxon has been associated with aggression parameters in our experiment. Bacterial taxa are included in the analysis if they occur in mean relative abundance of at least 1% in all samples.

Furthermore, *Clostridium sensu stricto 1* positively co-occurred in all samples with *Bifidobacterium* (Spearman rho = 0.76, *p<0*.001***), while *Bifidobacterium* was, in turn, negatively associated with the family Lachnospiraceae (Spearman rho = -0.52, *p<0*.001***) (Fig 5). The genus *Bifidobacterium* was associated with higher attack latency (Spearman rho = -0.47, *p<0*.05*), and the family Lachnospiraceae with a higher number of attacks (Spearman rho = 0.46, *p<0*.05*), in the samples received after the RI test (Fig 4). All pairwise correlations between combined rank score of aggression, alpha diversity and taxa occurrence are illustrated in S2 and S3 Figs in S1 File.

**Fig 6:**
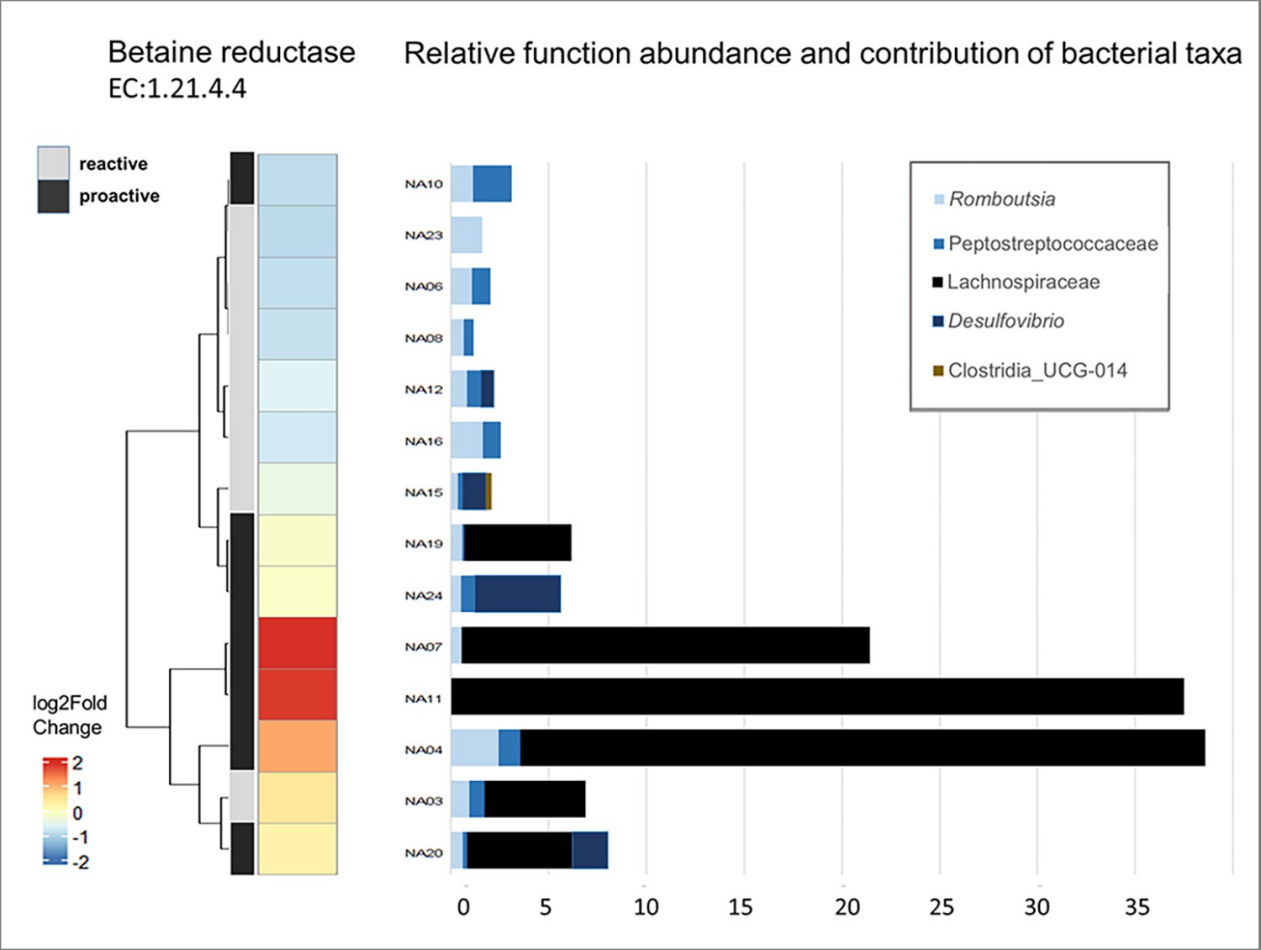
Differential abundance of betaine reductase in the rat microbiome. The relative abundance of betaine reductase in the microbiome of reactive and proactive rats is shown along with the contribution of specific bacterial taxa to the enzymatic function, expressed in percentage.

Finally, functional prediction analysis of the bacterial microbiome showed that the only enzymatic function which was significantly different between proactive and reactive rats was that of betaine reductase (Enzyme Commission Number EC 1.21.4.4). More specifically, the function showed a logarithmic two-fold change of 1.93 (adjusted p-value = 0.01), with the microbiome of proactive rats showing higher levels. When dividing our samples in two groups taken before and after the RI test, we detected a significant difference of betaine reductase enzymatic function only in rats after the RI test with a logarithmic two-fold change of 2.965483 (adjusted p-value < 0.001). Investigation of the bacterial taxa contributing to the presence of the betaine reductase enzymatic pathway showed that the family of Lachnospiraceae was the main contributor, followed by *Romboutsia* (Fig 6). Both taxa have beed associated with aggressive proactive rats either directly, in the case of Lachnospiraceae (Fig 4), or indirectly, in the case of *Romboutsia*, by co-occurring with other taxa indicative of proactive rats (Fig 5).

## Discussion

The individual differences in behavioral coping strategies and associated neuroendocrine and physiological (re)activity result from a complex bidirectional interaction between genetic background and environmental modulation [34] and the gut-brain axis reflects this bidirectional relationship [35]. In recent years, the gut microbiome has become an important player in understanding this interaction, as reviewed by Sarkar et al. [36]. Moreover, specific microbiome characteristics are associated with dominant or submissive behavior, which is subject to change when altering the microbiome [37]. The relationship between aggression and gut microbiota has been reviewed recently [38].

The main aims of the present study were to examine whether the pre-existing gut microbiota in GWT rats can predict a coping strategy with regard to aggression and/or whether the development of a specific coping strategy could shape the gut microbiota. In the present study, the level of aggressive behavior towards an unfamiliar intruder in the territory of a resident rat was used as a measure of coping strategy [12, 15, 39].

We hypothesized that an initially uniform microbiome in the group-housed breeding situation would change due to the RI experience. For that, longitudinal changes of the gut bacterial communities were studied by comparisons before and after the RI training and testing at a population level and pairwise for every individual. Furthermore, to investigate community differences between individuals assigned to different personalities during the RI test, we compared the microbiota structure of rats according to their demonstrated aggression levels.

We found that the bacterial communities changed after the RI experience, with alpha diversity significantly declining. This was observed for all personality groups and can be attributed to differences in housing conditions, due to the rats being initially co-housed with several individuals before being transferred to an environment with only one female for conducting the RI test. Furthermore, the beta diversity of the communities was significantly altered after the RI test.

No differences in alpha diversity were associated with differences in assigned personality. However, the phylogenetic diversity of the gut microbiota was negatively correlated with offensive behavior and the number of attacks in the RI test. This contrasts with studies on dogs [8, 9], where aggression was associated with higher alpha diversity. On the other hand, “calmness” scores in the collard peccary were positively related to bacterial evenness, suggesting a more homogeneous microbiome [40]. Similarly, the overall microbiota structure, as reflected by beta diversity estimates, did not differ between animal personalities. A closer look, however, revealed bacterial taxa associated with aggression.

### Microbiota members associated with aggression

In samples received before the RI training and testing, the relative abundance of the family Ruminococcaceae UCG-5 and the Gram-negative bacterium *cTPY-13* were negatively correlated with the subsequent aggressive behavior recorded in the RI. In a recent study, Ruminococcaceae UCG-5 has been associated with externalizing difficulties in children [41], while in an older study, the family had been associated with surgency in boys [42], a temperamental trait of early childhood, predictive of subsequent externalizing difficulties. In another recent study, the Gram-negative bacterium *cTPY-13* was related to depressive-like behavior in mice induced by exposure to deltamethrin [43]. Overall, data indicated that group-housed rats might have potential predispositions for a specific coping strategy.

In samples received after the RI test, *Clostridium sensu stricto 1* and *Blautia* were mainly associated with reactive rats. In a study with female rodents, *Clostridium sensu stricto 1* was associated along with *Bifidobacterium* to stress-resilient individuals [44]. In another study with mice, *Blautia* has been associated with less anxiety, again along with *Bifidobacterium* [45]. More specifically, transplants of *Blautia coccoides* resulted in reduced anxiety, while transplants of *Bifidobacterium infantis* resulted in less hyperactivity and less anxiety compared to controls [45]. Also, in two studies with dogs, *Blautia* was associated with low aggression together with *Lactobacillus* [9, 46].

As summarized in the previous paragraph, both phylotypes indicative of the reactive GWT rats gut microbiota, *Clostridium sensu stricto 1* and *Blautia*, have been associated with lower aggression along with lactic acid bacteria such as *Lactobacillus* and *Bifidobacterium*. *Bifidobacterium*, in particular, is a bacterial genus that has been frequently reported in gut microbiota-behavior studies as able to reverse aggressive behavior [38]. Also, in a study including mice with chronic social defeat stress, stress-resilient mice showed an increased abundance of *Bifidobacterium spp*. [47]. In our experiment, *Bifidobacterium* was both correlated with higher attack latency and highly co-occurred with *Clostridium sensu stricto 1*, suggesting that the impact of the microbiome on mood is a matter of community rather than a matter of individual taxa.

On the other hand, the genera *Alloprevotella* and *Anaerobiospirillum* were positively correlated to aggression. In a study in mice, *Alloprevotella* was found in higher abundance in individuals with lower chronic stress-induced depression-like behaviors due to the deactivation of the class B scavenger receptor CD36, which is involved in the cytotoxicity associated with inflammation [48]. Again, the strong co-occurrence of *Alloprevotella* with phylogenetically close phylotypes under the family Prevotellaceae in our dataset could stress the importance of the community effect on behavior.

### Predicted functions of microbiota members in association to aggression

Analysis of predicted enzymatic functions revealed only one enzyme with a significantly different abundance between reactive and proactive rats in our dataset. This enzyme, betaine reductase, catalyzes a chemical reaction resulting in producing betaine, or else trimethylglycine. Betaine is an amino acid derivative found in diverse organisms. Along with other -mostly beneficial- roles in health that have been tested on animal models [49], betaine as a supplement has been associated with higher power performance and higher testosterone levels in humans [50, 51], as well as with enhanced skeletal muscle differentiation in murine models [52]. Its presence in proactive, more aggressive rats after the RI test could indicate the effect of developing a coping strategy on the shaping of the gut microbiome.

The bacterial taxon that mainly contributed in the higher betaine reductase abundance in proactive rats was the family Lachnospiraceae. The relative abundance of Lachnospiraceae was significantly higher after the RI test and was associated with proactive rats attempting a higher number of attacks against the intruder in the RI test. What is more, the family was negatively associated with both *Clostridium sensy stricto 1* and *Bifidobacterium* that were indicative of reactive rats. We suggest that the association of the family with more aggressive rats, could be explained when taking into account its contribution in the betaine reductase function abundance. Furthermore, another bacterial genus that contributed to betaine reductase abundance, *Romboutsia*, cooccured with taxa related to proactive rats, even though it was not directly correlated with aggression.

### Importance of current results and study limitations

Most literature studies focus on the microbiome effects in socially defeated animals [5, 53, 54] or in animals with established “personalities” [7, 9], however, the present study differs in combining longitudinal changes of the microbiome before and after the development of a coping strategy. Microbiota differences before the development of a strategy could have a predictive value. Craddock *et al*., in a study aiming at predicting working dog behavior based on machine learning from microbiome features and species abundances, found a significant predictive capability of the microbiome for aggression, sociability, and motivation [8]. Furthermore, microbiota differences after developing a strategy can show how social experience can alter our gut microbiota, particularly when taking into account functional pathways which associate with certain behavioral traits.

On the other hand, as with all animal studies, the present one also shows some limitations. Apart from the social challenge of RI training and testing, rats have experienced additional changes in their social environment, including transitioning from group housing with male mates to individual housing with a female rat. The transition from group housing is expected to cause changes in the microbiome, regardless of which coping strategy rats adopt in the RI test, such as the observed reduction of alpha-diversity. Also, in group-housed rats, there can also be some degree of dominance as a predisposition for later coping strategies, which may be overshadowed by the co-housing associated exchange of microbiome. Furthermore, the RI test is a complex social experience, including stages such as co-habitation with a female, training, and defending territory against an intruder. Behavioral measurements for the assignment of a coping strategy are concentrated on the final stage of defense against an intruder. Therefore, further investigation would be useful to specify the contribution of all different aspects of the RI paradigm to the observed effects on the microbiome. Finally, the present study was based on one cohort of rats. Although these rats were not necessarily siblings, they may share some genetic background.

## Conclusions

Our data indicate that the social experience of the Resident Intruder test alters the gut microbiome in GWT rats. Moreover, some aspects of the microbiome obtained before the RI experience may be of predictive value for the subsequently developed coping strategy. The microbiome changes over the RI experience could also be associated with specific coping strategies developed by rats during the test, since different groups of bacterial phylotypes were related to different levels of aggression and to different predicted functions of the microbiome.

## Supporting information

S1 File Supplementary file. Bacterial community composition and pairwise rank correlations between aggression parameters and main bacterial taxa.
(PDF)

S1 Data (XLSX)
