# Supplementary material for: Aggression shapes the gut microbiome; a study in rats
Source: PLoS One. 2024 Oct 22;19(10):e0312423. doi: 10.1371/journal.pone.0312423 (PMC11495597; doi:10.1371/journal.pone.0312423)
Supplement: S1 File — Bacterial community composition and pairwise rank correlations between aggression parameters and main bacterial taxa. (PDF) [file pone.0312423.s001.pdf]

Supplementary File

Aggression shapes the gut microbiome; a study in rats

Anna Voulgari-Kokota[1,2], Joana Falcao-Salles[1], Regien G. Schoemaker[1]

[1] Groningen Institute for Evolutionary Life Sciences (GELIFES), University of Groningen, P.O. Box 11103, 9700 CC, Groningen, The Netherlands

[2] Laboratory of Microbiology, Wageningen University, 6700 EH, Wageningen, The Netherlands

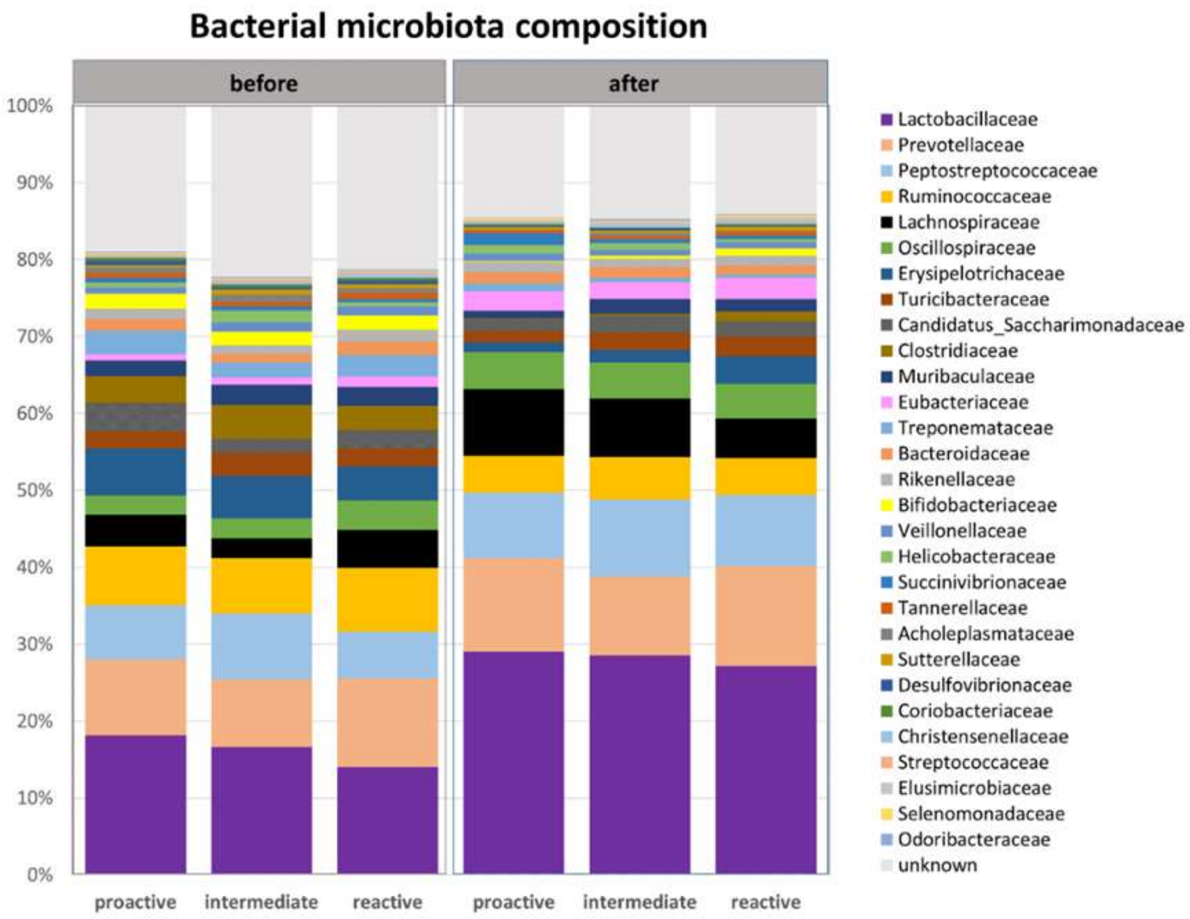

Figure S1. Bacterial community composition at the family level of GWT rat gut samples for three personality types (proactive, intermediate, reactive) before and after the resident-intruder test. Each barplot summarizes seven biological replicates. Bacterial families are included if the summed relative abundance of the phylotypes assigned to them is detected at a percentage of at least 5% in the whole dataset.

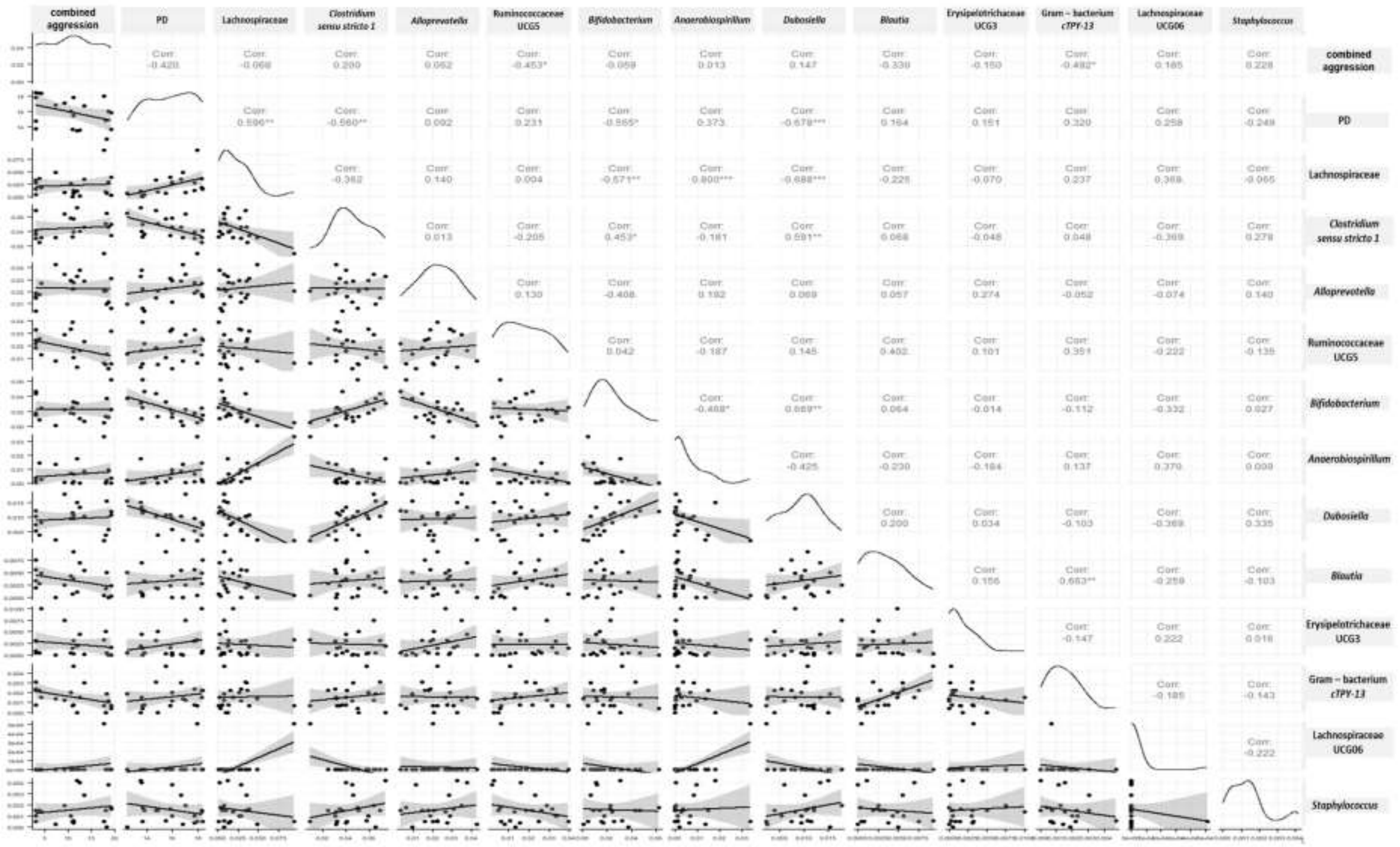

Figure S2. Pairwise rank correlations between combined aggression demonstrated in the Resident – Intruder (RI) test, the phylogenetic alpha diversity and the relative abundance of selected bacterial taxa before the RI test. Combined aggression is the z-score calculated from attack latency, number of attacks and percentage time spent on offensive behavior. The bacterial taxa included are the ones significantly associated with aggression either before or after the RI test. p=0.5., p<0.05*, p<0.01**, p<0.001***

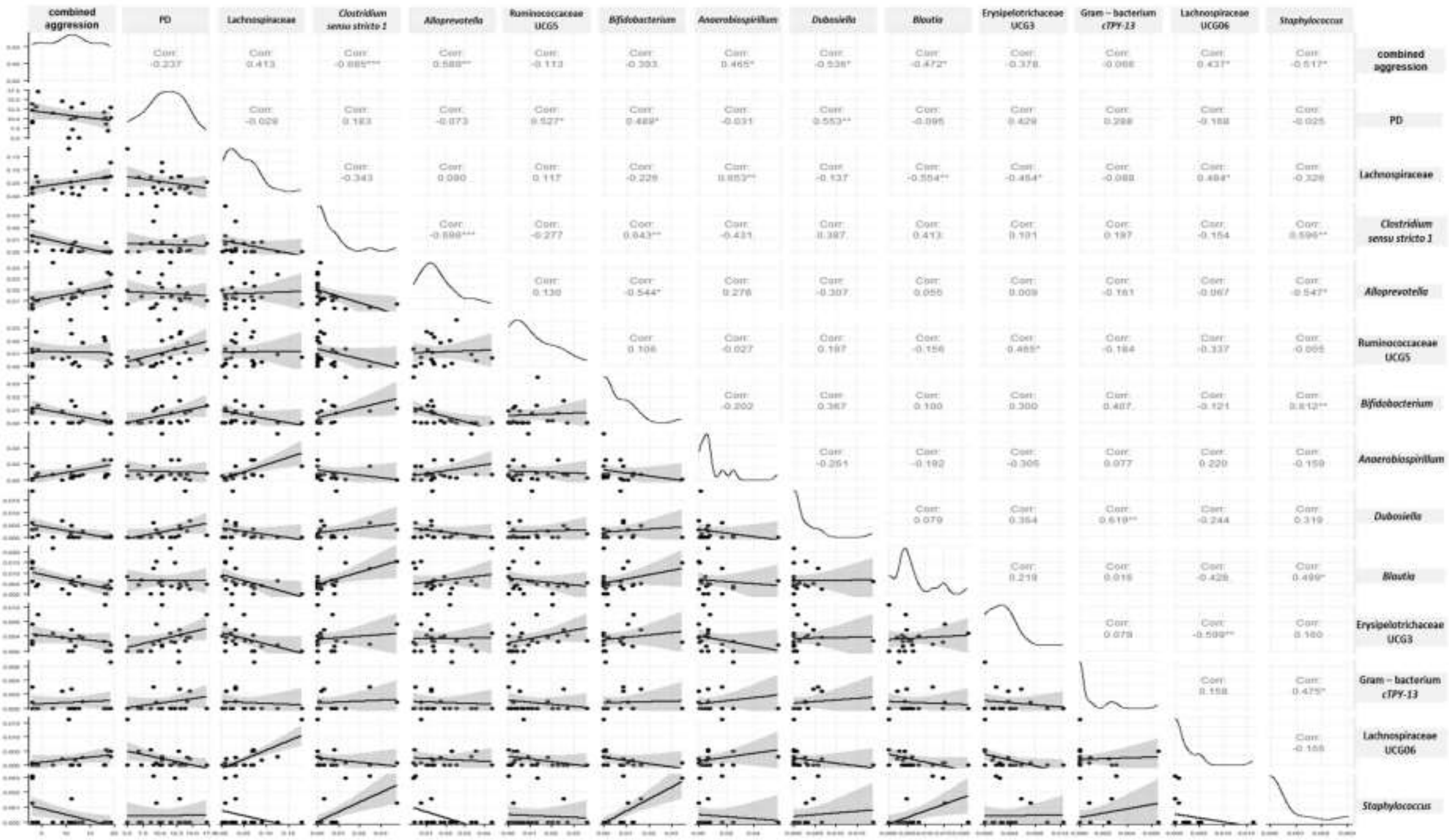

Figure S3. Pairwise rank correlations between combined aggression demonstrated in the Resident – Intruder (RI) test, the phylogenetic alpha diversity and the relative abundance of selected bacterial taxa after the RI test. Combined aggression is the z-score calculated from attack latency, number of attacks and percentage time spent on offensive behavior. The bacterial taxa included are the ones significantly associated with aggression either before or after the RI test. p=0.5., p<0.05*, p<0.01**, p<0.001***
